# Recent advances in amidyl radical-mediated photocatalytic direct intermolecular hydrogen atom transfer

**DOI:** 10.3762/bjoc.21.100

**Published:** 2025-06-27

**Authors:** Hao-Sen Wang, Lin Li, Xin Chen, Jian-Li Wu, Kai Sun, Xiao-Lan Chen, Ling-Bo Qu, Bing Yu

**Affiliations:** 1 Medical School, Huanghe Science and Technology College, Zhengzhou, 450006, PR China; 2 College of Chemistry, Zhengzhou University, Zhengzhou 450001, PR China; 3 Institute of Chemistry, Henan Academy of Sciences, Zhengzhou 450002, PR China; 4 National Engineering Research Center of Low-Carbon Processing and Utilization of Forest Biomass, Nanjing Forestry University, Nanjing 210037, PR China

**Keywords:** amidyl radicals, C–H, HAT reagents, hydrogen-atom-transfer, late-stage functionalization

## Abstract

In recent years, amidyl radicals have emerged as highly efficient and versatile reagents for hydrogen atom transfer (HAT) in photocatalytic reactions. These radicals display exceptional selectivity and efficiency in abstracting hydrogen atoms from C–H, Si–H, B–H, and Ge–H, positioning them as invaluable tools in synthetic chemistry. This review summarizes the latest advancements in the photocatalyzed generation of amidyl radicals as HAT reagents, with a particular emphasis on their role in the intermolecular HAT process. We highlight key developments, mechanistic insights, and emerging strategies that harness the unique reactivity of amidyl radicals in the selective functionalization of a variety of substrates.

## Introduction

**Figure 1:**
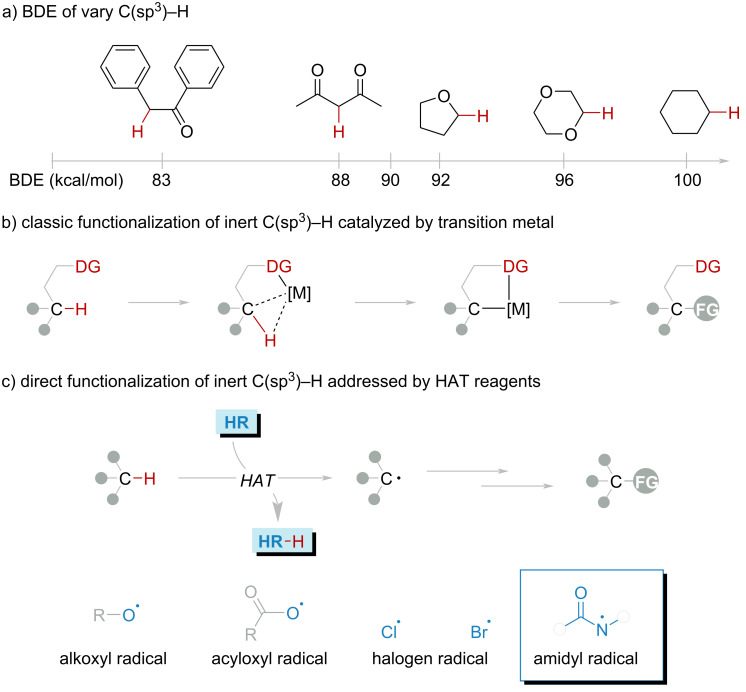
(a) BDE of C–H. (b) Direct functionalization of C–H catalyzed by transition-metal. (c) Direct functionalization of C–H via HAT process.

C–H bonds are the predominant chemical bonds in organic compounds, and their direct conversion can rapidly and efficiently increase the complexity and functionality of organic molecules. On the other hand, C–H bonds exhibit low reactivity due to their relatively high bond dissociation energy (BDE) (Figure 1a). Therefore, the direct functionalization of C–H bonds is extremely challenging [1–5].

In recent decades, transition-metal-catalyzed C–H bond functionalization demonstrated a decent methodology of organic synthesis. These elegant strategies presented powerful C–H bond transformation toolkits (Figure 1b) [6–8]. One of the exceptions to the perfection is the pre-functionalization of substrates. Current catalytic methodologies predominantly rely on substrate prefunctionalization through directing group (DG) incorporation, inevitably necessitating covalent DG-metal coordinative anchoring. This prerequisite fundamentally compromises both atomic efficiency and synthetic practicality, thereby imposing fundamental constraints on the catalytic system's intrinsic sustainability and operational scalability [9–10]. Moreover, a high temperature and additive oxidants are generally required, which would limit the substrate scope.

The hydrogen atom transfer (HAT) process has emerged as a powerful avenue for addressing these challenges, leveraging the HAT reagents to selectively abstract hydrogen atoms from these C–H bonds and directly functionalize these bonds via radical reactions (Figure 1c) [11–18]. This approach involves HAT reagents abstracting hydrogen atoms from C–H bonds to generate highly reactive C-centered radicals, which can subsequently form C–C or C–heteroatom bonds. The incorporation of HAT strategies into the functionalization of C–H bonds represents a significant advancement in synthetic organic chemistry for their high atom economy and step economy.

**Figure 2:**
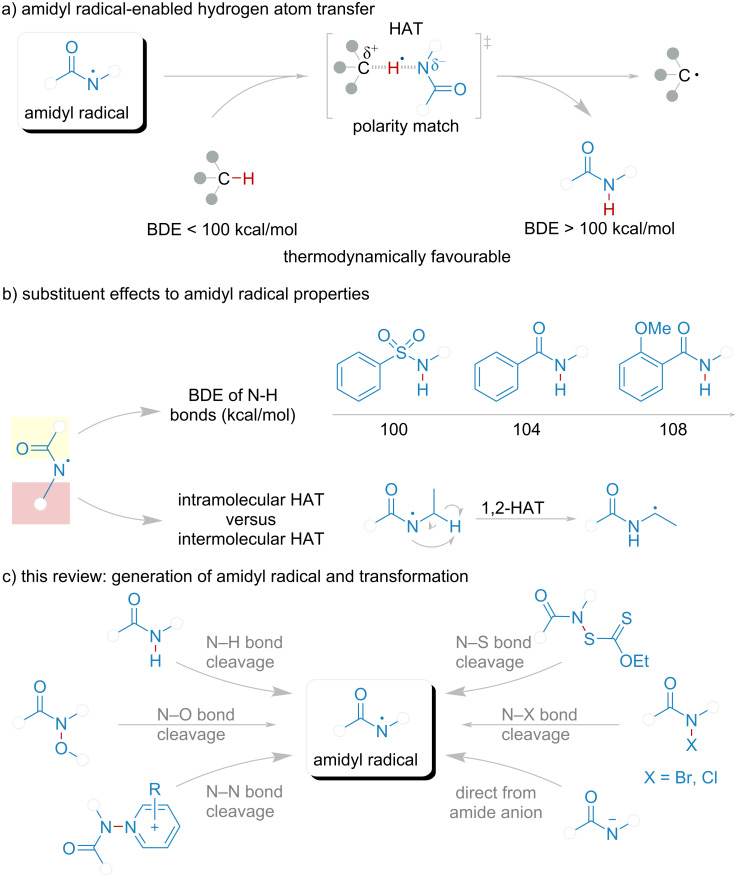
(a) Amidyl radical-enabled hydrogen atom transfer. (b) Substituent effects to amidyl radical properties. (c) This review: generation of amidyl radical and transformation.

HAT reagents (HR), including alkoxyl, acyloxyl, halogen radicals, and amidyl (Figure 1c) [19–27], serve as key species for the HAT process. These HR were generated from different HAT reagent precursors (HRP) in a variety of strategies. Among these, amidyl radical HRPs have gained significant attention in recent years due to their ease of HRP synthesis and the relatively green chemistry of generating amidyl radicals. Amidyl radicals offer several advantages that enhance their applicability in organic synthesis:

1) The BDE of amidyl N–H bond is more than 105 kcal/mol, relative to the bond (C–H, Si–H, B–H, and Ge–H) which BDE is lower than 100 kcal/mol (Figure 2a) [28–30]. Almost 5 kcal/mol difference between two species could spontaneously undergo a HAT process. That also justifies the selectivity and efficiency of amidyl radical serving as HAT reagent.

2) Recent research indicated a critical correlation between electronic effects and activation energy modulation during transition state formation. Specifically, donor/acceptor electronic configurations in the substrate could either stabilize or destabilize the transient hybrid state, thereby thermodynamically governing the energy barrier for intermolecular HAT progression. When the partial positive and negative charges of two species can be stabilized by the electronic effects, these species are considered to be polarity matched during the HAT process. Conversely, when there is a polarity mismatch, the HAT process is likely to be impeded (Figure 2a). C–H bonds predominantly prefer to be nucleophilic, which smoothly facilitates the HAT process with amidyl radical. This effect is also called a polarity match [31–39].

3) Considering the electronic effect, modifying the substituent of the N atom could tune the property of HAT capability (Figure 2b) [40–43]. The electron-withdrawing groups could stabilize the charge of the N-centered radical during the HAT process by decreasing the charge density [44]. Notably, the BDE of N–H in the corresponding amide might be too low to ensure a spontaneous HAT process due to the electronic effect of the substituent. When introducing electron-donating groups to address this contradiction, another vital impact arises, the intramolecular HAT would take place. The amidyl radical would abstract a hydrogen atom from the nearest C–H, i.e., 1,2-HAT. Taken all these together, the substituent group should be decently modified.

In recent years, photocatalysis has been widely adopted due to its green and efficient nature [45–51]. The generation of amidyl radical is implemented by HRP. Six different methods (Figure 2c), which have been developed for visible-light mediated reactions, could generate amidyl radicals from HRP: (a) direct single-electron oxidation of amide HRP in the presence of photocatalyst and a base via a proton-coupled electron transfer (PCET) process by the cleavage of the N–H bond; (b) single-electron reduction of HRP catalyzed by photocatalyst via a single-electron transfer (SET) process by the cleavage of the N–O bond; (c) direct homolytic cleavage of weak N–S or N–X bonds in HRP initiated in the presence of visible light; (d) the intersystem crossing (ISC) of S_1_ to T_1_ state directly from the amide anion. This review is organized by bond cleavage type, offering a deep insight in the development of novel methods for amidyl radical-mediated photocatalytic direct intermolecular hydrogen atom transfer.

**Figure 3:**
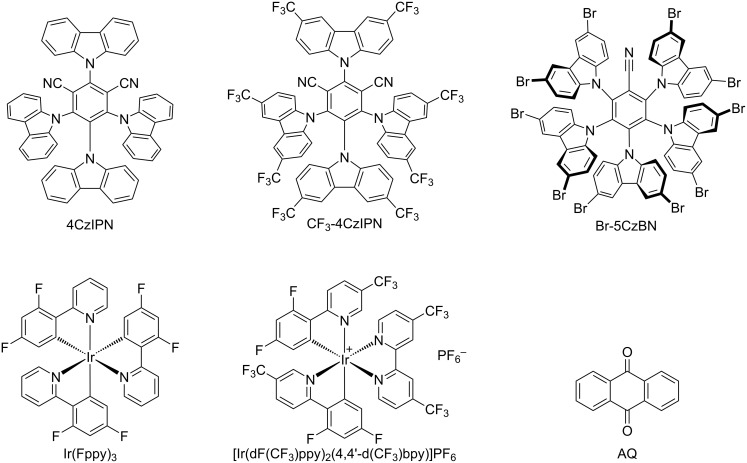
Representative photocatalysts discussed in this review.

Although, amidyl radicals employed in many reactions as HAT reagents via heating conditions have been summarized in several studies [52–58]. To advance the research of direct functionalization via HAT processes and the development of green chemistry in photocatalysis, this review will focus on the generation of amidyl radicals and reaction mechanisms and highlight the photocatalyzed reaction characteristics. This review aims to provide researchers with a systematic understanding and strategic toolkit, thereby propelling the development of direct functionalization of C–H, B–H, Si–H, and Ge–H techniques in modern organic synthesis. Most of the photocatalysts used in this review are listed in Figure 3.

## Review

### Amidyl radical from N–H bond cleavage

*N*-Alkylbenzamide constitutes the primary structural unit of this class of compounds. The structures of these compounds are relatively simple and readily synthesizable. In these photocatalytic systems, direct single-electron oxidation of the amide HRP occurs in the presence of a photoredox catalyst and a base via a proton-coupled electron transfer process [59–69]. Following this process, the corresponding amidyl radical abstracts a hydrogen atom from the substrate, resulting in the conversion of the amidyl radical back to *N*-alkylbenzamide. This pathway creates a complete cycle in synchrony with the photocatalytic cycle, thereby allowing these HRPs to be consistently employed for catalytic equivalence.

**Scheme 1:**
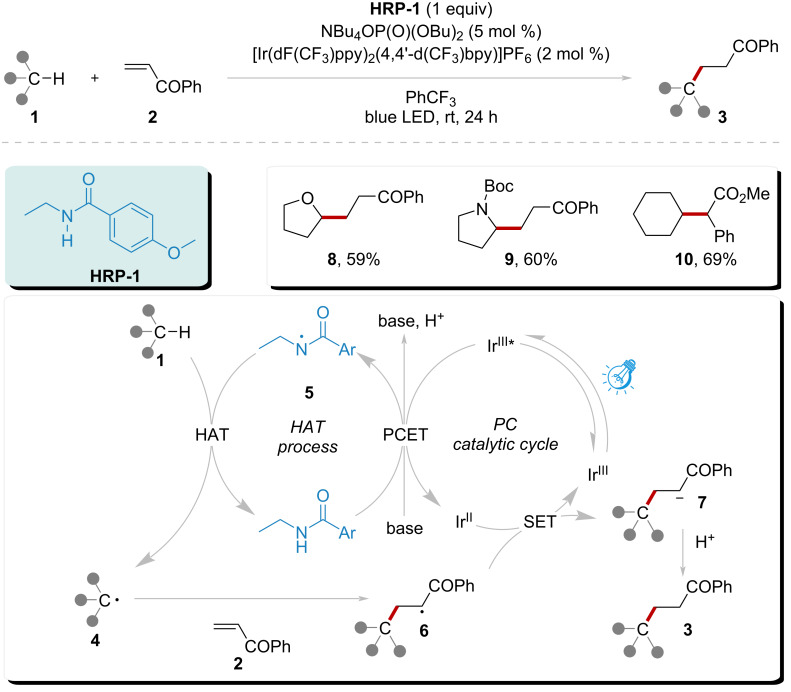
Alkylation of C(sp^3^)–H catalyzed by amidyl radical under visible light.

In 2016, Knowles’ group independently developed an oxidative photocatalytic system capable of directly generating amidyl radicals from *N*-ethyl-4-methoxybenzamide, utilizing the photocatalyst [Ir(dF(CF_3_)ppy)_2_(4,4'-d(CF_3_)bpy)]PF_6_ in combination with a base (NBu_4_OP(O)(OBu)_2_) (Scheme 1) [59]. The generation of amidyl radical **5** involved a stepwise PCET process catalyzed by the combined effect, in the presence of photocatalyst and the base. Subsequently, amidyl radical **5** abstracted a hydrogen atom from substrate **1**. This HAT process returned the amidyl radical **5** to **HRP-1**, enabling the continuation of the HAT cycle in synchronization with the photocatalytic cycle. The resulting radical **4** then underwent Giese addition with activated alkenes, leading to the formation of products **8**, **9**, and **10** with 59%, 60%, and 69% yields. This powerful and efficient toolkit effectively overcame the limitations of intramolecular HAT processes.

**Scheme 2:**
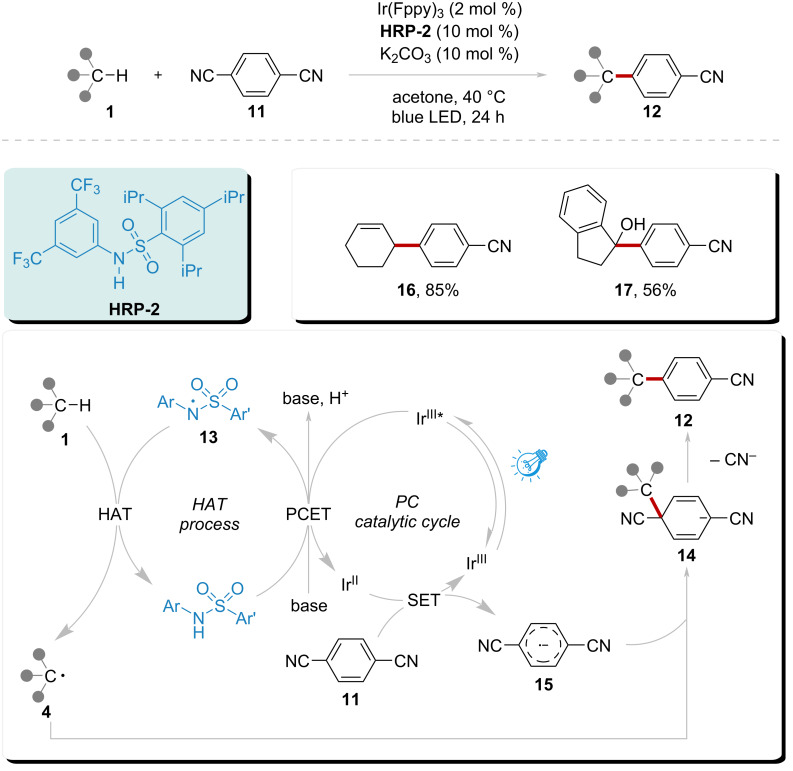
Direct heteroarylation of C(sp^3^)–H catalyzed by amidyl radical under visible light.

Building on this strategy, Kanai’s group reported a novel HAT method employing a new radical precursor in an oxidative photocatalytic system in 2018 (Scheme 2) [70]. Through a similar oxidative pathway, amidyl radical **13** was generated directly from the amide **HRP-2**, facilitating a smooth HAT process with substrate **1** while simultaneously regenerating **HRP-2**. The resulting radical **4** then participated in an addition reaction with radical anion **15**. The radical anion **15** was reduced by the photocatalyst Ir(Fppy)_3_ from the reagent **11**. The resulting anion **14** underwent aromatization to release a nitrile anion, subsequently yielding product **12**. This strategy also successfully produced products **16** and **17** with yields of 85% and 56%, respectively, from cycloalkenes and alcohols.

**Scheme 3:**
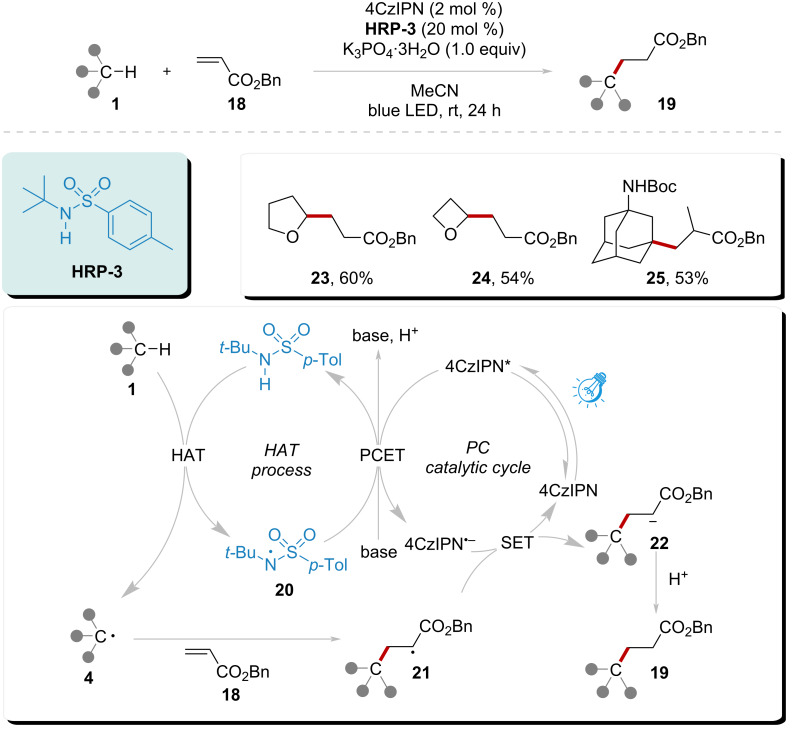
Alkylation of C(sp^3^)–H catalyzed by amidyl radical and metal-free photocatalyst under visible light.

To eliminate the need for noble metal photocatalysts in the system, Duan’s group employed 2,4,5,6-tetra-9*H*-carbazol-9-yl-1,3-benzenedicarbonitrile (4CzIPN) as a metal-free photocatalyst (Scheme 3) [71]. This system initiated the formation of amidyl radical **20** from **HRP-3** through a PCET process, involving the oxidation of excited 4CzIPN* and deprotonation by a base. The resulting amidyl radical **20** smoothly abstracted a hydrogen atom from the substrate via a HAT process, generating a radical **4**. This C-centered radical subsequently underwent Giese addition with activated alkenes, resulting in the formation of radical **21**. Radical **21** then oxidized the photocatalyst radical anion to its ground state while simultaneously generating anion **22**. Ultimately, anion **22** yielded product **19** through protonation. This system demonstrated good applicability, achieving yields of 53% to 60% for products **23**, **24**, and **25**.

**Scheme 4:**
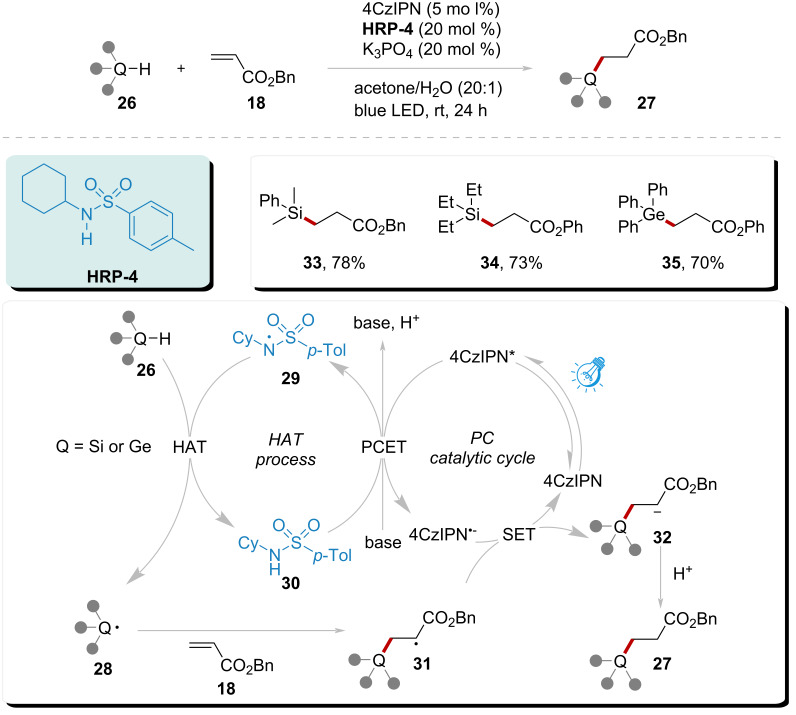
Alkylation of C(sp^3^)–H, Si–H, and Ge–H catalyzed by amidyl radical under visible light.

To further investigate the scope of substrates, Selvakumar’s group employed **HRP-4** in combination with 4CzIPN (Scheme 4) [72]. This system examined the applicability of Si–H and Ge–H bonds through a HAT process. As seen in previous strategies, **HRP-4** was converted into amidyl radical **29** in the presence of 4CzIPN and a base via a PCET process. Radical **29** subsequently engaged in a HAT process with substrate **26**, generating either a Si radical or a Ge radical **28**. Following this, radical **28** underwent Giese addition with activated alkenes. The reduction of species **31** was efficiently promoted by the PC radical anion. The resulting anion **32** ultimately produced product **27** through protonation. This system demonstrated the significant HAT capability of amidyl radical **29**, as evidenced by the synthesis of products **33**, **34**, and **35** with yields reaching 70% to 78%.

### Amidyl radical from N–N bond cleavage

*N*-Amidopyridinium salts are known to undergo SET reduction, leading to the formation of amidyl radicals. Hong’s group has made significant advances in the cleavage of N–N bonds in recent years. Through SET reduction of *N*-amidopyridinium salts to generate amidyl radicals, Hong’s group has accomplished various remote functionalizations of C–H bonds via 1,5-hydrogen atom transfer processes [73–76].

**Scheme 5:**
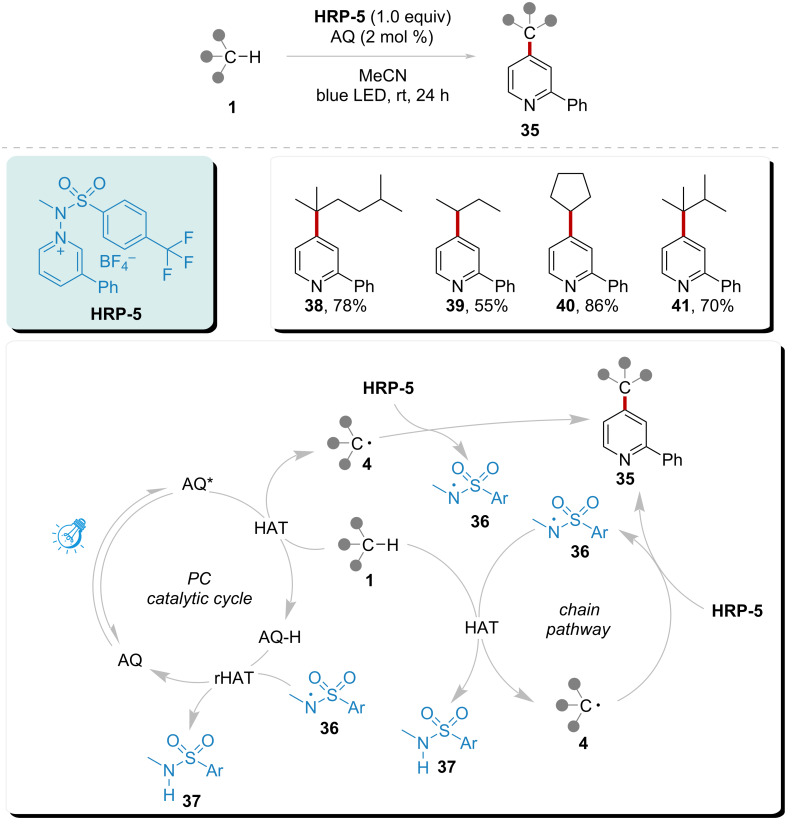
Direct heteroarylation of C(sp^3^)–H catalyzed by synergistic promotion of amidyl radical and photocatalyst, under visible light.

In 2021, Hong’s group reported a HAT combined with a reverse hydrogen atom transfer (rHAT) system (Scheme 5) [77]. By utilizing anthraquinone (**AQ**) as the HAT photocatalyst, activated **AQ** was able to abstract a hydrogen atom from substrate **1**. The addition of the corresponding radical **4** to **HRP-5** facilitated the release of amidyl radical **36**, which simultaneously produced the final product **35**. Amidyl radical **36** was capable of abstracting hydrogen atoms from both substrate **1** and **AQ**–**H**. The HAT process between substrate **1** and amidyl radical **36** initiated a chain reaction pathway leading to the formation of product **35**. Conversely, the rHAT process between amidyl radical **36** and **AQ**–**H** allowed for the regeneration of the photocatalyst and the completion of the catalytic cycle. Amidyl radical **36** played a central role in this photocatalytic system. This strategy demonstrated good chemical selectivity for the functionalization of pyridine and alkanes, resulting in 55% to 86% yields of products **38**, **39**, **40**, and **41**, respectively.

### Amidyl radical from N–O bond cleavage

In 2022, Alexanian’s group demonstrated the homolytic cleavage of the N–O bond using *N*-(*tert*-butyl)-*O*-(1-phenylvinyl)-phenylhydroxyamide as a HAT reagent [78–79]. This compound was capable of initiating the formation of amidyl radicals through visible light activation. Although their controlled experiments showed that this method was effective, the use of heating conditions resulted in a higher yield of the corresponding products. This advancement prompted scientists to explore alternative pathways for generating amidyl radicals, as an alternative to the traditional SET reduction of the N–O bond [80–82]. The SET reduction is able to produce amidyl radicals and oxygen anions in the presence of photocatalysts activated by visible light. Two representative cases illustrating this approach were reported in 2023.

**Scheme 6:**
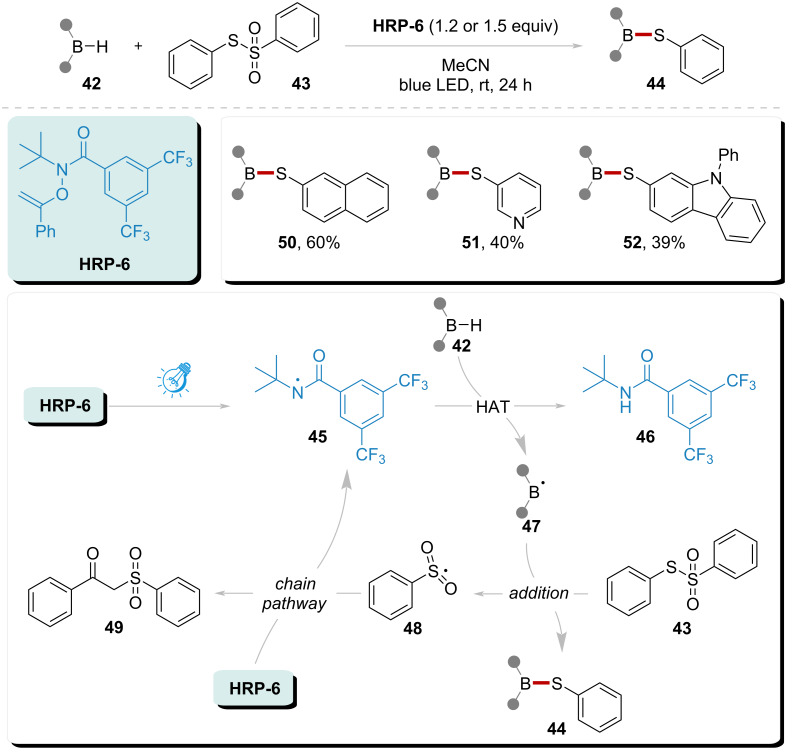
Direct B–H functionalization of icosahedral carboranes catalyzed by amidyl radical under visible light.

Building upon the experiments conducted by Alexanian’s group, Yan’s group extended the applicability of carborane as a HAT substrate (Scheme 6) [83]. Initially, under optimized conditions, **HRP-6** was employed to generate amidyl radical **45**, which subsequently participates in the HAT process with the carborane substrate. This process results in the formation of borone radical **47**, accompanied by amide **46**. The resultant radical **47** can be intercepted by species **43**, simultaneously releasing radical **48** and product **44**. Radical **48** reacts with **HRP-6**, leading to the regeneration of amidyl radical **45**, the release of byproduct **49**, and the initiation of a chain reaction pathway. Notably, this system could give rise to the formation of the highly applied value products **50**, **51**, and **52**, with the 39% to 60% yields. The work by Yan demonstrated the HAT capabilities of amidyl radical **45** and significantly broadened the substrate scope of amidyl radical-enhanced photocatalytic systems.

The reduction of the N–O bond through traditional SET processes is effectively illustrated by *N*-(acyloxy)phthalimides [84]. These compounds preferentially undergo SET reduction, resulting in the cleavage of the N–O bond. Typically, this cleavage generates an amidyl anion and an O radical [5,85–88]. Conversely, it is also possible for the N–O bond to produce an amidyl radical alongside an O anion.

**Scheme 7:**
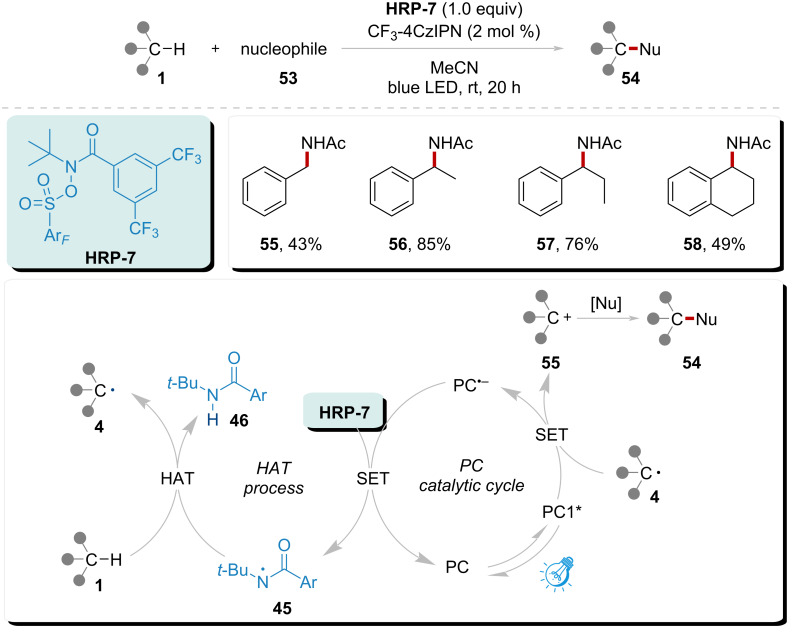
Nucleophilic amination of C(sp^3^)–H enabled by amidyl radical under visible light.

In 2023, Doyle’s group reported a novel system initiated by an off-cycle reductive quenching of the activated CF_3_-4CzIPN* species, leading to the generation of a ground state photocatalyst radical anion (Scheme 7) [89]. This radical anion subsequently underwent SET reduction of **HRP-7**, resulting in the liberation of amidyl radical **45**. The amidyl radical **45** efficiently abstracted a hydrogen atom from substrate **1**, yielding radical **4** and byproduct amide **46**. Furthermore, the resultant radical **4** was oxidized by the excited photocatalyst, resulting in the concurrent generation of the carbon cation **55**. This cation was subsequently trapped by a nucleophile, leading to the formation of product **54**. This system demonstrated a broad applicability for the general nucleophilic amination of benzylic C–H bonds. The substrate's scope and selectivity were exemplified by the satisfactory yields of products **55**–**57**, and **58**, which achieved yields of 43–85%.

**Scheme 8:**
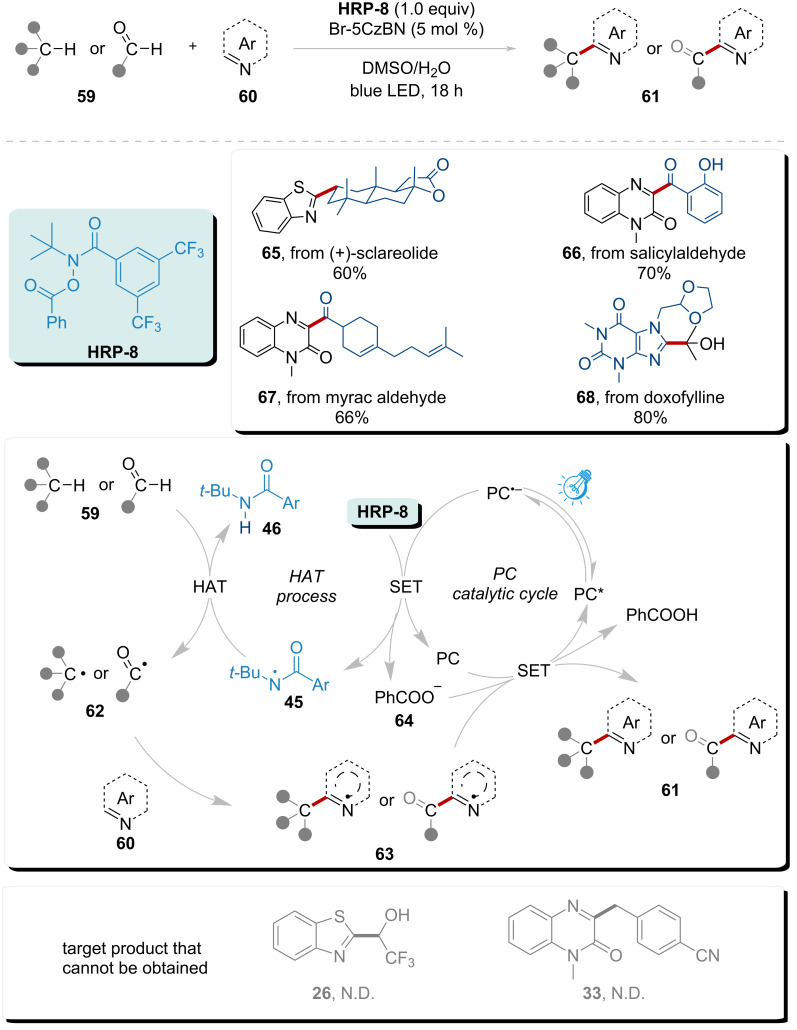
Direct heteroarylation of C(sp^3^)–H and C(sp^3^)–H without the presence of strong bases, acids, or oxidants.

Inspired by these previous work, Yu’s group devised a new photocatalyzed system catalyzed by a newly designed photocatalyst Br-5CzBN. This robust strategy implements direct heteroarylation of C(sp^3^)–H and C(sp^3^)–H without the presence of strong bases, acids, or oxidants (Scheme 8) [90]. The reaction is initiated by SET reduction of **HRP-8** via excited photocatalyst Br-5CzBN*, subsequently generating HAT reagent **45**, O-anion **64**, and Br-5CzBN^+•^. HAT reagent **45** engages a HAT event with the substrate, converting it into the byproduct **46** and generating a carbon-centered radical **62**. Species **62** is trapped by heteroarene **60**, leading to the formation of the intermediate **63**. This intermediate **63** undergoes SET and proton transfer with the assistance of O-anion **64** and the Br-5CzBN^+•^ radical cation, delivering the final product **61** and regenerating photocatalyst Br-5CzBN. **HRP-8** functions as an oxidizing agent, facilitating the generation of a highly active HAT reagent, while the O-anion **64** serves as a base. This elucidates the rationale behind the self-sufficiency of this photocatalytic system, obviating the need for supplementary oxidants and bases, thereby enabling broad substrate adaptability. This streamlined approach demonstrates significant potential for extended utility in pharmaceutical late-stage functionalization (LSF), particularly evidenced by the synthetically valuable yields (60–80%) obtained for structurally diversified target molecules **65**–**68** under optimized reaction conditions. In these cases, the high polarity of the radical intermediates of CF_3_CH_2_OH and 4-methylbenzonitrile, combined with the poor solubility of adamantane, may explain the possible reasons behind the reaction process [91].

### Amidyl radical from N–S bond cleavage

The work of Alexanian’s group has significantly advanced the field of organic synthesis over recent years, particularly in the area of N–S bond homolytic cleavage [92–94]. Initial studies demonstrated that high-temperature conditions were required to facilitate this reaction; however, such extreme conditions limited the practical applicability of the reactions. To address this limitation, Alexanian's research has shifted toward the principles of green chemistry, utilizing visible light to achieve mild reaction conditions. This approach has not only enhanced the feasibility of the reactions but has also led to the establishment of a comprehensive platform for C–H functionalization through the introduction of the highly versatile xanthyl functional group.

**Scheme 9:**
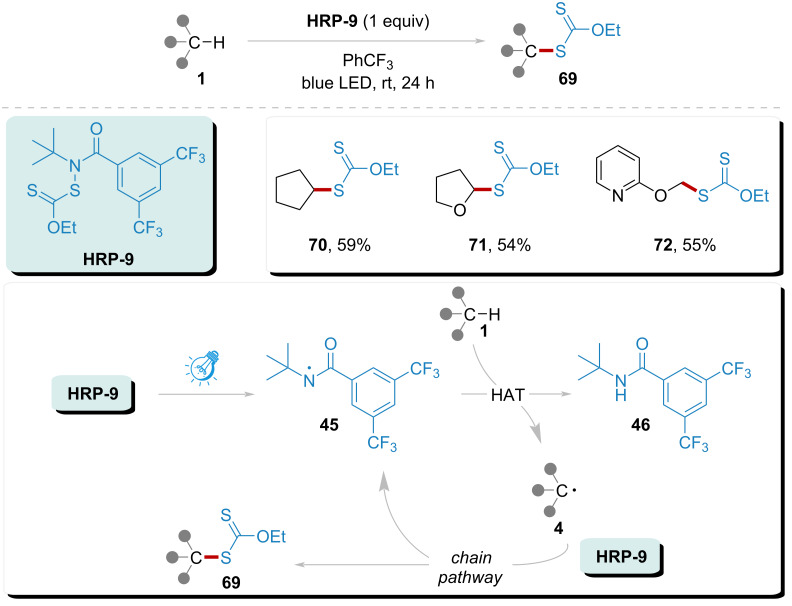
Xanthylation of C(sp^3^)–H addressed by amidyl radical under visible light.

In 2016, Alexanian’s group successfully implemented a method for the direct xanthylation of C–H bonds with high selectivity and efficiency (Scheme 9) [95]. This process is initiated through a visible light-triggered chain reaction, involving the homolytic cleavage of **HRP-9**. The liberation of amidyl radical **45** facilitates hydrogen atom abstraction from the substrate, resulting in the formation of radical **4** and the concurrent generation of byproduct **46**. Subsequent trapping of radical **4** by **HRP-9** leads to the generation of product **69**. This methodology demonstrates a broad substrate scope and exhibits significant synthetic utility, particularly for the generation of products **70**, **71**, and **72** with yields ranging from 54% to 59%.

**Scheme 10:**
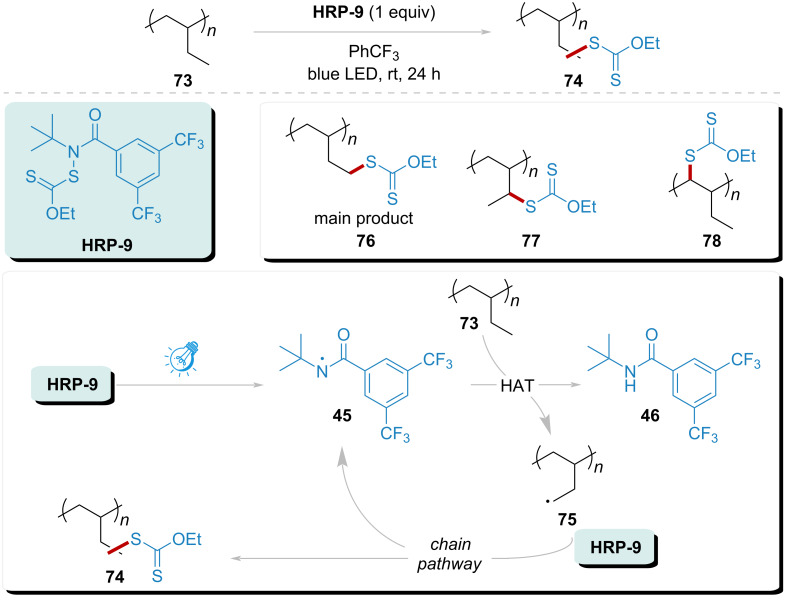
Xanthylation of C(sp^3^)–H in polyolefins addressed by amidyl radical under visible light.

In an effort to evaluate the practicality of visible light-promoted xanthylation, Alexanian’s group conducted a regioselective C–H xanthylation of polyolefins (Scheme 10) [96]. Building on previous findings, the **HRP-9** undergoes homolytic N–S cleavage, yielding amidyl radical **45**. This radical effectively abstracts a hydrogen atom from the polyolefin substrate, demonstrating notable regioselectivity. The xanthylation reaction preferentially generates main products **76**, and byproducts (**77** and **78**). Furthermore, the successful implementation of this methodology facilitates the production of a diverse range of functionalized polyolefins, showcasing the applicability of this xanthylated polyolefin in various reactions, including trifluoromethylthiolation, polymer grafting, Michael addition, and epoxide opening.

### Amidyl radical from N–X bond cleavage

Direct halogenation of C–H bonds is of significant value in organic synthesis. Introducing a bromine or chlorine atom into aliphatic C–H bonds with high site selectivity and efficiency poses a formidable challenge. Traditional strategies for the halogenation of aliphatic C–H bonds typically rely on biomimetic iron-catalyzed oxidation systems that require electrophilic heterocycles. These limitations hinder the broader application of such systems.

Alexanian’s group modified amidyl radical precursors by incorporating halogen atoms, transforming them into bifunctional reagents. The HAT component of amidyl radical precursors was facilitated by amidyl radicals, while halogenation was promoted by the introduced halogen atom [26].

**Scheme 11:**
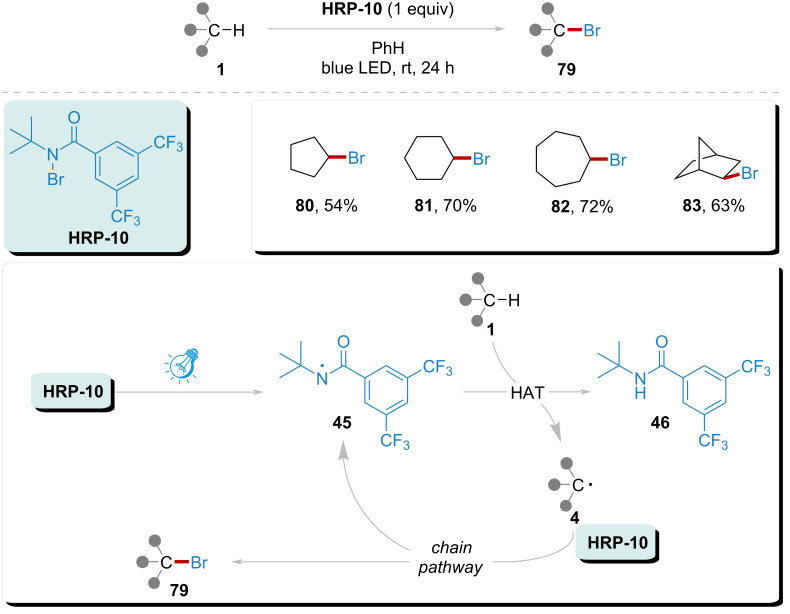
Site-selective C(sp^3^)–H bromination implemented by amidyl radical under visible light.

In 2014, Alexanian’s group reported a site-selective aliphatic C–H bromination utilizing modified HRP as both the bromination reagent and HAT reagent (Scheme 11) [25]. Initiated by visible light, **HRP-10** underwent homolytic cleavage of the N–Br bond, generating amidyl radical **45**. This amidyl radical subsequently participated in a HAT process with the aliphatic substrate **1**, leading to the formation of radical **4** and byproduct amide **46**. The corresponding radical **4** was then trapped by **HRP-10**, thereby triggering a chain reaction that regenerated amidyl radical **45**. This system effectively examined the site selectivity of aliphatic C–H bromination, yielding products **80**–**82**, and **83** with 54% to 63% yields at selective positions. This bromination system provided a mild reaction environment suitable for aliphatic C–H bonds.

**Scheme 12:**
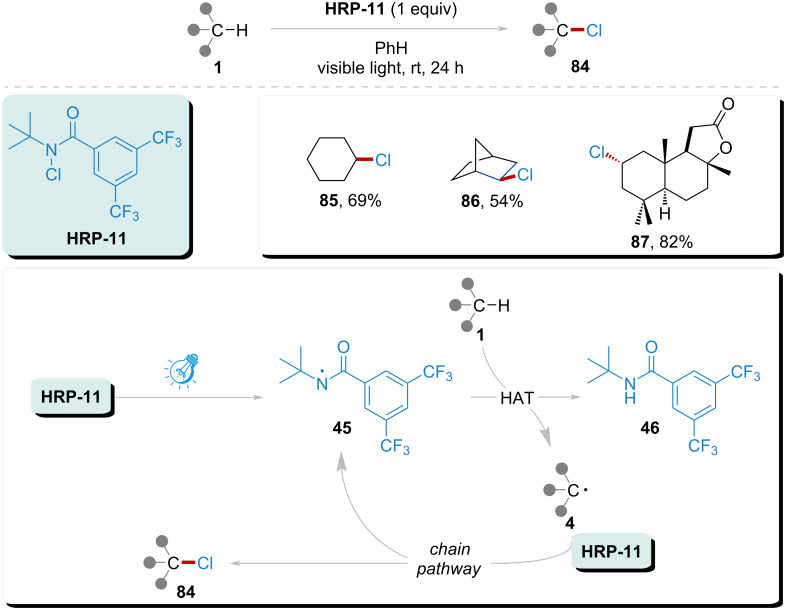
Site-selective chlorination of C(sp^3^)–H in natural products implemented by amidyl radical under visible light.

In 2016, Alexanian’s group reported a chlorination method for aliphatic C–H bonds with high site selectivity to expand the halogenation capabilities and applicability of their system (Scheme 12) [97]. Consistent with previous experiments, the reaction was initiated by visible light, generating amidyl radical **45** from **HRP-11**. The resulting radical **45** abstracted a hydrogen atom from substrate **1**, simultaneously generating radical **4**. Subsequently, radical **4** was trapped by **HRP-11**, leading to the formation of chlorinated product **84**. In this system, monochlorinated products were obtained with good selectivity, evidenced by yields of 69% and 54% for products **85**, and **86**, respectively. Notably, the natural product sclareolide underwent chlorination with an impressive selectivity, achieving an 82% yield for the product **87**.

### Amidyl radical from amide anion

**Scheme 13:**
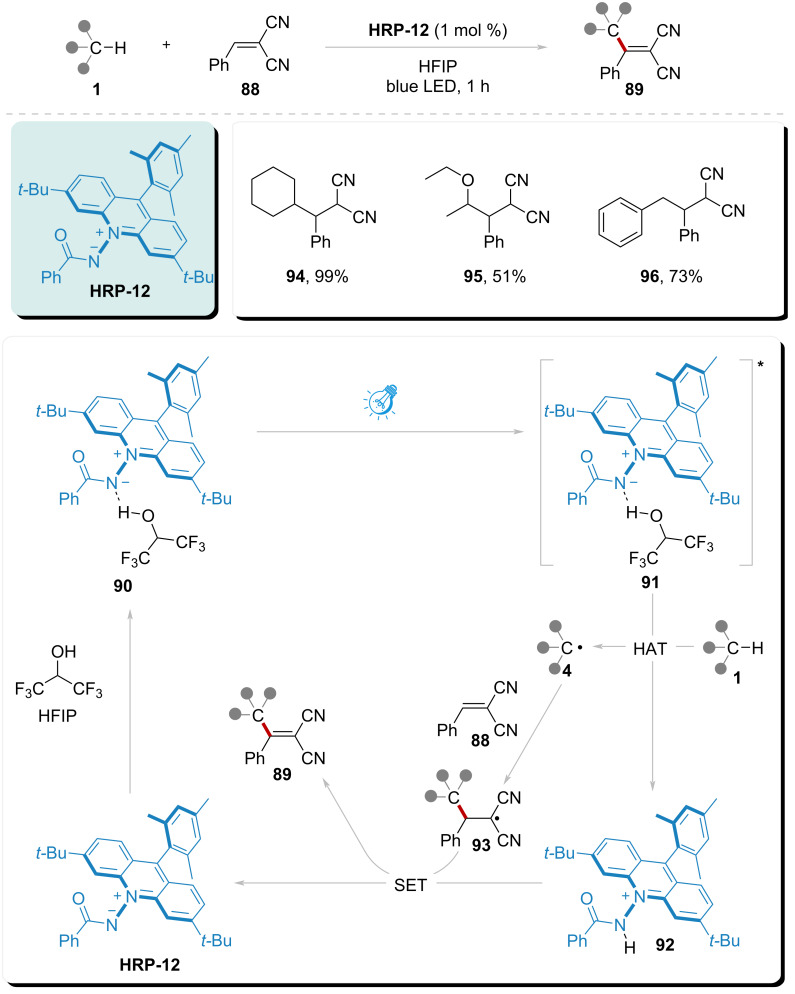
Alkylation of C(sp^3^)–H catalyzed by amidyl radical photocatalyst under visible light.

In 2024, Ooi and colleagues established a pivotal advancement in catalytic methodology through the rational design of zwitterionic acridinium amidates. These photoactive amidyl radical precursors demonstrated exceptional HAT reactivity, enabling efficient functionalization of unactivated C–H bonds under mild irradiation conditions (Scheme 13) [98]. The mechanistic pathway initiates with ground-state complexation **90** between **HRP-12** and HFIP via hydrogen bonding. Following visible-light excitation of **90**, intersystem crossing (ISC) from the S1(LE) to T2 state generates the catalytically competent triplet excited state **91**. This N-centered radical species subsequently abstracts a hydrogen atom from substrate **1** through HAT, producing an α-amido-acridinyl radical intermediate **92** and a substrate-derived carbon-centered radical **4**. Radical **4** undergoes regioselective addition to the acceptor, forming transient radical adduct **93**. A concomitant SET from **92** to **93** generates a carbanion, which undergoes either solvent-mediated protonation or direct proton transfer from the acridiniumamide, ultimately delivering product **89** while regenerating the zwitterionic **HRP-12** catalyst.

This catalytic platform demonstrated exceptional site selectivity in aliphatic C–H bromination under ambient temperature and visible-light irradiation, achieving site-selective bromination (products **94**–**96**) in 51–99% yields across electronically differentiated positions. The system's operational mildness and functional group tolerance highlight its suitability for late-stage functionalization of complex aliphatic architectures.

## Conclusion

In this review, we highlight recent advances in the use of visible light to enhance amidyl radical-mediated direct intermolecular HAT for the functionalization of C–H, Si–H, Ge–H, and B–H bonds. These robust strategies hold the promise of the direct functionalization of these bonds through high selectivity, efficiency, and a stepwise approach.

In the presence of amidyl radicals, hydrogen atoms are directly abstracted from C–H, Si–H, Ge–H, and B–H bonds, leading to the formation of corresponding radicals. We summarize and emphasize notable pioneering experiments in this area. Switchable amidyl radicals provide an effective toolkit for completing hydrogen atom transfer processes. Transitioning from noble metal photocatalysts to organic photocatalysts and from HAT reagents to bifunctional reagents, these remarkable photocatalytic systems have inspired innovations across various fields of organic synthesis methodology.

Despite significant research achievements of amidyl radicals in the photocatalytic transformation of C(sp³)–H, C(sp²)–H, S–H, Ge–H, and B–H bonds, they still face considerable challenges in the application of hydrogen abstraction from electron-deficient C–H bonds due to their inherent polarity. Furthermore, amidyl radicals encounter difficulties in regioselectivity when applied to structurally complex C–H substrates, which limits their utility in modifying intricate molecular architectures. Future advancements are anticipated through structural modifications of amidyl radicals aimed at optimizing their polarity via electronic effects, which may enhance their effectiveness in hydrogen abstraction from electron-deficient substrates. Additionally, strategies to optimize steric effects could improve their regioselectivity in hydrogen abstraction from complex substrates.

Crucially, structural optimization of HRP components could potentially overcome current mechanistic limitations, establishing a generalized platform for hydrogen atom transfer (HAT)-enabled direct functionalization. This advanced methodology would demonstrate unprecedented versatility across diverse bond activation challenges, particularly in C(sp³)–H, C(sp²)–H, S–H, Ge–H, and B–H bond transformations. The proposed system architecture emphasizes synergistic reagent cooperation rather than isolated component performance, representing a paradigm shift in photoredox catalysis design principles.

## Data Availability

Data sharing is not applicable as no new data was generated or analyzed in this study.
